# The British Medical Library Association

**Published:** 1909-01-23

**Authors:** 


### THE BRITISH MEDICAL LIBRARY ASSOCIATION.

At a meeting of those interested in medical libraries held at Leeds on Saturday, January 9, it was decided unanimously to form an Association of Medical Libraries, and a Provisional Committee was appointed to draw up the constitution and rules. Professor Osler (Oxford) was invited to become the first President, and Professor Walker Hall (Bristol) and Mr. Cuthbert E. A. Clayton (Librarian Manchester Medical Society) were asked to undertake the duties of temporary secretaries. The following are some of the objects of the Association :—(1) Intercourse of those interested in medical library work and the discussion of matters associated with the fostering and care of libraries. (2) Diffusion of information as to the branches of medical literature specially catered for at different centres, and as to the value of the various books and new periodicals which are issued from time to time. (3) The promotion of measures whereby a larger number of practitioners in each centre may be induced to utilise the library facilities of each district. (4) The consideration of matters connected with the present rapid increase of periodicals, publications of books, etc. (5) The opening up of better chances of advancement for library assistants. By such means there would be an inducement to parents to put their sons to such an occupation, and the librarians would be able to have better material for training. (6) The exchange of duplicate books and periodicals. When the Association is in working order it is hoped that a certain number of the libraries interested will join together to form a circle for the loan of their research and other literature.

